# Affective Latent Representation of Acoustic and Lexical Features for Emotion Recognition

**DOI:** 10.3390/s20092614

**Published:** 2020-05-04

**Authors:** Eesung Kim, Hyungchan Song, Jong Won Shin

**Affiliations:** 1 AI R&D Team, Kakao Enterprise, 235, Pangyoyeok-ro, Bundang-gu, Seongnam-si, Gyeonggi-do 13494, Korea; chris.ekim@kakaoenterprise.com; 2 School of Electrical Engineering and Computer Science, Gwangju Institute of Science and Technology, 123 Cheomdan-gwagiro, Buk-gu, Gwangju 61005, Korea; shchan420@gist.ac.kr

**Keywords:** emotion recognition, conditional adversarial autoencoder, latent representation

## Abstract

In this paper, we propose a novel emotion recognition method based on the underlying emotional characteristics extracted from a conditional adversarial auto-encoder (CAAE), in which both acoustic and lexical features are used as inputs. The acoustic features are generated by calculating statistical functionals of low-level descriptors and by a deep neural network (DNN). These acoustic features are concatenated with three types of lexical features extracted from the text, which are a sparse representation, a distributed representation, and an affective lexicon-based dimensions. Two-dimensional latent representations similar to vectors in the valence-arousal space are obtained by a CAAE, which can be directly mapped into the emotional classes without the need for a sophisticated classifier. In contrast to the previous attempt to a CAAE using only acoustic features, the proposed approach could enhance the performance of the emotion recognition because combined acoustic and lexical features provide enough discriminant power. Experimental results on the Interactive Emotional Dyadic Motion Capture (IEMOCAP) corpus showed that our method outperformed the previously reported best results on the same corpus, achieving 76.72% in the unweighted average recall.

## 1. Introduction

Emotions play an important role in successful communication among humans [1], and thus, more attention is given to recognize, interpret, and process emotional information effectively [2,3,4]. Human emotion can be expressed by various means, but two of the main cues are what and how people say something. There have been many research works to recognize human emotion from the speech signal based on acoustic features [5,6,7,8,9,10,11,12,13,14,15,16,17,18], lexical features [19,20], or both of them [21,22,23,24,25,26,27,28,29,30,31,32].

One of the main research topics for speech emotion recognition is to find appropriate features that discriminate different emotions [6,7,8,9,10,11,12,13,14,15,16,17,18,19,20,21,22,23,24,25,26,27,28,29,30,31,32,33]. Rozgic et al. [21] suggested statistical functionals of the low-level descriptors (LLD) and a model-based feature set based on Mel frequency cepstral coefficients (MFCC) scored against emotion-dependent Gaussian mixture models (GMM) as acoustic features, bag-of-words (BOW) representations based on the linguistic inquiry and word count, as well as the general inquirer lexicon for lexical features. Jin et al. [22] used three types of acoustic features including LLD, Gaussian supervectors, and bag-of-audio-words. These acoustic features are combined with an e-vector, which adopts a salience information weighting scheme and BOW. Gamage et al. [23] suggested another weighting scheme to capture the emotional salience from spoken content and verbal gestures using either word or phoneme level transcripts.

Recently, deep learning approaches have been incorporated in speech emotion recognition and brought performance improvement [9,10,11,12,13,14,15,16,17,24,25,26,27,28,29,30,31,32,33]. In [24], a deep neural network (DNN) classifier was adopted for which the input consisted of acoustic features extracted from Mel frequency spectral coefficients using a convolutional neural network (CNN)-long short-term memory (LSTM), other acoustic features obtained by a DNN based on the LLD, and lexical features extracted as outputs of CNNs from words and parts-of-speech tags. In [25], another deep hierarchical multimodal structure was proposed incorporating an attention mechanism to focus on informative words and attentive audio frames for each modality and word-level fusion. In [28], DNN-based bottleneck acoustic features with statistical functionals were used as input features along with two types of lexical features including word2vec and affective lexicon-based dimensions with the term frequency-inverse document frequency weighting scheme. This combination of features greatly improved the performance of the speech emotion recognition, but the classifier was a classical feed-forward DNN. There also have been research works that obtained features from the acoustic information by applying generative models including autoencoders [10], adversarial autoencoders (AAEs) [11], conditional AAEs (CAAEs) [9], variational autoencoders (VAEs) [10], conditional VAEs [10], adversarial variational Bayes [11], and generative adversarial networks [12]. Among them, the CAAE [9] showed potential capability to find very low-dimensional latent representations of acoustic features relevant to emotion classification, but the performance was slightly lower than that using raw features.

**Figure 1:**
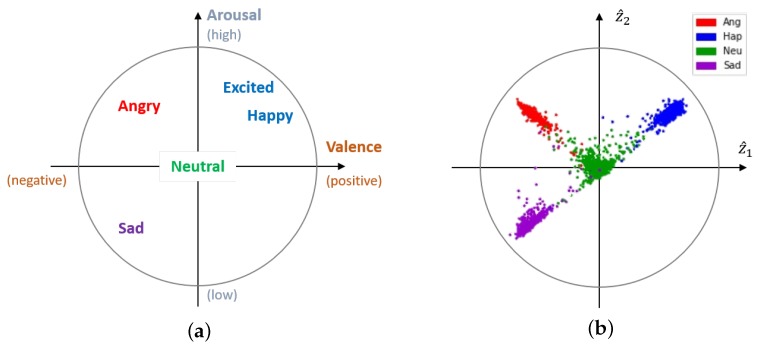
Valence-arousal space of the circumplex model (**a**) and the distribution of the learned latent vectors for the training set of the Interactive Emotional Dyadic Motion Capture (IEMOCAP) dataset (**b**).

In this paper, we propose a speech emotion recognition system that captures emotion-related underlying factors using a CAAE [34] from acoustic and lexical information. First, we extract various utterance level features from the acoustic and lexical information such as DNN bottleneck acoustic features, LLD, word2vec, affective lexicon-based features, and BOW. Then, a CAAE finds two-dimensional affective latent representation from the combined features, which can be set to be similar to a vector in the valence-arousal space (VA space) in the circumplex model in psychology [35] as shown in Figure 1. By setting the proper prior distribution of the latent representation, the classification can be done by simply checking the signs of the latent vector components without any well-trained classifier. The proposed method achieved 76.72% in the unweighted average recall (UAR) on the Interactive Emotional Dyadic Motion Capture (IEMOCAP) dataset [36], which confirmed that the CAAE was suitable for emotion recognition if the input features had enough discriminant power.

## 2. Adversarial Autoencoder-Based Emotion Recognition Using Acoustic and Lexical Features

**Figure 2:**
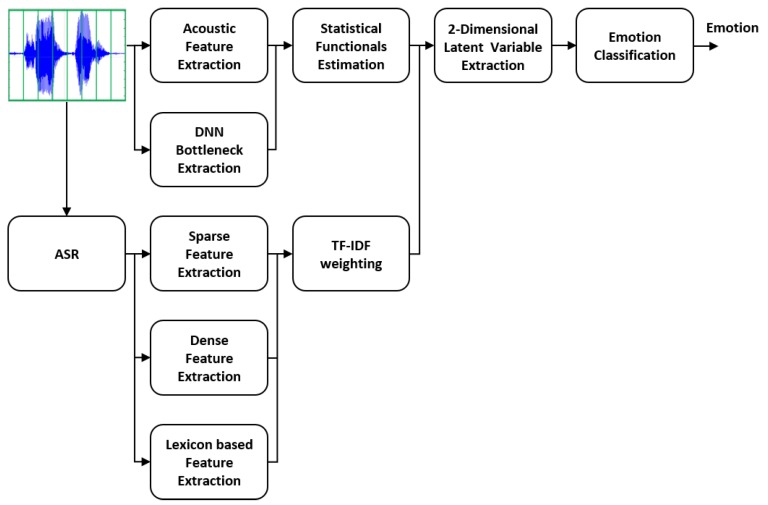
Overview of the proposed multimodal emotion recognition framework integrating the acoustic and lexical features.

One of the popular models for emotion in psychology is the circumplex model [35], which represents various emotions in a VA space as shown in Figure 1a. Valence indicates the pleasure of a speech, while the arousal means the emotional activation level. We adopted a CAAE to extract latent factors related to emotions from the acoustic and lexical features, which can be configured to have an analogy with the emotion representation in the VA space. Figure 1b shows the scatter plot of the latent vectors extracted by the CAAE for the training dataset, which exhibited a clear relationship with the circumplex model. Figure 2 illustrates an overview of the proposed multimodal emotion recognition architecture. We first extracted LLD and DNN bottleneck acoustic features at the frame-level. Utterance-level acoustic features were obtained by applying statistical functions to frame-wise features. Frame-level linguistic information was modeled using a sparse representation, a distributed representation, and affective lexicon-based features. Then, we derived the utterance-level lexical features by applying a weighting called the term frequency-inverse document frequency (TF-IDF). These utterance-level acoustic and linguistic features were fed into a CAAE, which produced two-dimensional underlying factors for emotional speech classification. The emotional class was simply decided according to in which quadrant the latent vector fell.

### 2.1. Acoustic and Lexical Feature Set

Many previous works [5,6,7,8,9,10,11,12,13,14,15,16,17,18] attempted to recognize the emotions of speakers using acoustic features from speech signals. Meanwhile, several approaches have been proposed to recognize the emotions of users using only the lexical features obtained from textual dialogue [19,20]. As the meaning of the words itself has affective information, the lexical features extracted from “what is said” would have complementary information to the acoustic features, which is on “how it is said.” Utilizing both acoustic and lexical features brought performance improvements in many previous studies [21,22,23,24,25,26,27,28,29,30,31,32].

In this paper, we used the acoustic and lexical features similar to those in our previous work [28]. As for the acoustic features, the acoustic features obtained from the bottleneck layer of a DNN used in [28] were used along with low-level features such as voicing probability and fundamental frequency estimated by PEFAC [37], MFCC, and Mel filter bank energies features. On the other hand, lexical information was utilized in the form of two distributed vector representations, BoW and word2vec, and affective lexicon-based features. BoW converts text to a vector by counting the number of appearances for each word in the dataset, and word2vec uses a continuous bag-of-words (CBoW) neural net model to predict a target word from the context words surrounding it across a fixed size context window [38]. As for the affective lexicon-based features, we used the Affective Norms for English Words (ANEW) lexicon [39], which contains 13,915 English words with scores in three affect-related dimensions with a value from one to 10 evaluated by humans.

The utterance-level acoustic features were derived from the frame-level features applying statistical functions [28]. The utterance-level lexical features were constructed from word-level lexical features such as BOW, word2vec, and lexicon-based ANEW dimensions through the TF-IDF weighting scheme in a similar manner as [28].

### 2.2. Extraction of Emotion-Relevant Latent Vectors Using a Conditional Adversarial Autoencoder

**Figure 3:**
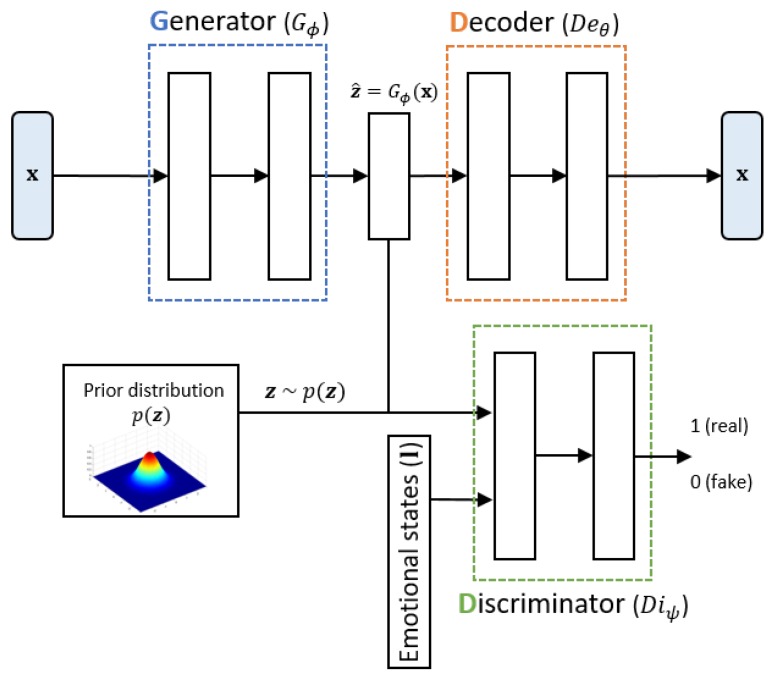
Architecture of the conditional adversarial autoencoder.

In the proposed system, a CAAE was adopted to map the combined utterance-level acoustic and lexical features to low-dimensional latent representation to discover underlying factors relevant to speech emotion classification. The architecture of the CAAE is shown in Figure 3. The CAAE consisted of three networks, which were the generator (or encoder) *G*, decoder De, and discriminator Di. The object function of the CAAE can be expressed as:

$$\begin{array}{ccc} \min_{\theta ,\phi }\max_{\psi } & E_{x∼p_{d}\left(x\right)}[d\left(x,De_{\theta }\left(G_{\phi }\left(x\right)\right)\right]+E_{z∼p(z)}\left[\log Di_{\psi }\left(z,l\right)\right] & \end{array}$$

(1) $$\begin{array}{cc} +E_{x∼p_{d}\left(x\right)}\left[\log\left(1-Di_{\psi }\left(G_{\phi }\left(x\right),l\right)\right)\right] & \end{array}$$

where p(z) is a desired prior distribution, $p_{d}\left(x\right)$ is the data distribution, and d(.) is the two-norm distance function. The neural networks *G*, De, and Di were parametrized with ϕ, θ, and ψ, respectively. The first term tried to make the output of De as close to the input $x\in R^{d}$ as possible. The second and third term represented *G* trying to make the output $ẑ\in R^{n}$ follow the prior distribution of the latent variable $z\in R^{n}$ and Di trying to discriminate z sampled from the prior distribution and **ẑ** obtained as an output of *G* given an additional condition $l\in R^{c}$, which was a one-hot vector representing the emotion label. The label acted as a switch that selected a specific part of the prior distribution according to the emotion class. After the training, only the *G* network was used to extract low-dimensional latent variables **ẑ** from the input feature vector.

**Figure 4:**
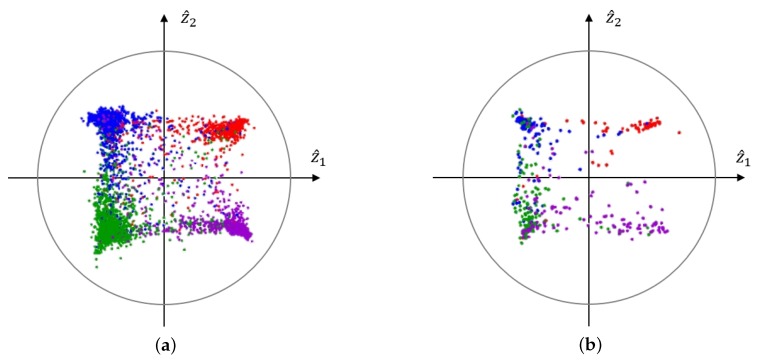

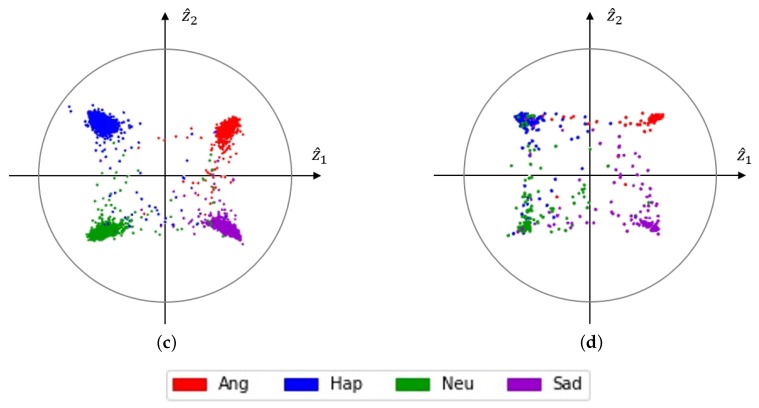
The distribution of the learned latent vectors for (**a**) the training set with the acoustic feature only, (**b**) the test set with the acoustic feature only, (**c**) the training set with both acoustic and lexical features, and (**d**) the test set with both acoustic and lexical features.

Training of the CAAE was carried out by alternating two phases, which were the reconstruction and regularization phases. In the reconstruction phase corresponding to the first term of the cost Equation (1), the autoencoder updated the generator and the decoder to minimize the reconstruction error. In the regularization phase related to the second and third terms in the cost Equation (1), the adversarial network first updated the discriminator to differentiate the z sampled from the prior distribution and the **ẑ** generated by the generator. The adversarial network then updated the generator to capture the prior distribution to confuse the discriminator. In the experiment, we selected the dimension of the latent variable, *n*, as two and used a four-mixture bivariate GMM as the prior distribution. Each mode of the GMM corresponded to one of the four emotion classes with equal probability, i.e., $p\left(z\right)∼\sum_{k=1}^{4}\frac{1}{4}N\left(\mu_{k},\Sigma_{k}\right)$ where $\mu_{k}$, $\Sigma_{k}$ denote the mean vector and the covariance matrix for each emotion, respectively. The mean vectors could be placed to match the VA space of circumplex model as shown in Figure 1, but we could classify the emotion without any sophisticated classifier when we let $\mu_{1}={\left[\begin{array}{cc} 1 & 1 \end{array}\right]}^{T},\mu_{2}={\left[\begin{array}{cc} 1 & -1 \end{array}\right]}^{T},\mu_{3}={\left[\begin{array}{cc} -1 & -1 \end{array}\right]}^{T}$, and $\mu_{4}={\left[\begin{array}{cc} -1 & 1 \end{array}\right]}^{T}$, as in Figure 4, which maximized the shortest distance between the mean vectors, resulting in better performance (The confusion matrices with various configurations are available at https://mspl.gist.ac.kr/ser_confusionmatrix/confusion_matrix.html).

It is noted that the configuration in Figure 1 that matched the VA space may be suitable for the estimation of the valence and arousal or the classification of more emotions. The covariance matrices were set to be identical for all classes, i.e., $\Sigma_{k}=diag\left\{{\left[\begin{array}{cc} 0.1 & 0.1 \end{array}\right]}^{T}\right\}$. The performance was not very sensitive to the dimension of the latent space or the location of the mean vectors.

## 3. Experiments

Experiments were performed for the IEMOCAP dataset [36] to demonstrate the effectiveness of our approach. In total, the dataset was about 12 h of audiovisual data (speech, video, facial motion capture), which consisted of dyadic interactions between professional female and male actors. The dataset was divided into 5 sessions, in which two actors performed a scripted play and improvised speech for each session. Three evaluators annotated each utterance in the dataset with the categorical emotion labels. In order to match the experimental condition with the previous studies [21,22,23,25], we used emotional utterances for which at least two out of three annotators gave the same emotion label among angry, happy, neutral, and sad. The happy and excitement classes were merged into the happy class to balance the data distribution between classes, resulting in 1103 angry, 1636 happy, 1708 neutral, and 1084 sad utterances. The resultant number of utterances was 5531 in total, and the average duration was 4.5 s.

The experiments were conducted in a leave-one-speaker-out cross-validation scheme to focus on speaker-independent emotion recognition. The test data for each of the 10-fold cross-validation session consisted of the utterances spoken by one specific speaker, while the training data became the utterances from the other 9 speakers. The performance was assessed using the weighted average recall (WAR) and the UAR. The WAR is the ratio of the total number of correctly predicted test samples and the total number of test utterances, while the UAR is defined as the accuracy per class averaged over all classes so that the accuracy for each class has the same importance regardless of the number of test samples in the class.

The proposed CAAE model consisted of the encoder, generator, and discriminator, each of which had 2 hidden layers of 1024 rectified linear units [40]. The activation function for the output layer of the discriminator was hyperbolic tangent. The dropout rate [41] in each hidden layer was 0.5. We applied early stopping when the CAAE model was trained over 100 epochs and both the loss of generator and reconstruction were no longer lowered for 5 epochs. The learning rates for the encoder, decoder, and discriminator were 0.001, 0.001, and 0.0002, respectively. The DNN for the bottleneck feature extraction was constructed to estimate the emotional label from the LLD with the structure of 55-256-256-23-256-4 [28]. The DNN for the utterance-level emotion classification had three hidden layers with 1024 units and was regularized using the dropout rate of 0.5.

In Table 1, the emotion classification performances with a DNN classifier using only acoustic features are shown for the proposed and previously published acoustic features [5,7,8,13,14,21,22,28], in which BN represents bottleneck features extracted using a DNN. We can see that the system using both the bottleneck features and LLD outperformed the previously published acoustic features. Applying CAAE to the acoustic features to extract two-dimensional latent vectors did not improve the performance of the emotion recognition, as reported in [9]. Table 2 shows the performances of the emotion recognition systems with lexical features only. Among the four features in the published papers [21,22,23,28], the concatenation of the word2vec and affective lexicon-based features in [28] exhibited the best performance. Incorporating BOW on top of the features in [28] did not improve the results, although the performance of the emotion recognition system with both acoustic and lexical features could be improved with BOW features. Like the case of the acoustic features, the CAAE-based two-dimensional representation showed slightly worse performance.

The performances of the proposed and previously reported emotion recognition systems [21,22,23,25,28] that utilized both the acoustic and lexical features are summarized in Table 3. We can see that the proposed system using CAAE-based affective latent representation gave the best results in both the WAR and UAR. The WAR for the proposed system with a DNN classifier was 74.37%, while the previously best performance reported for the same database was 72.7% [25], and the UAR was 76.91% when the previously best result was 74.31% [28]. Moreover, with the prior distribution indicated in Section 2.2 and illustrated in Figure 4, we could obtain 76.72% of the UAR by just checking the signs of the components of the extracted latent vector without any sophisticated classifier (denoted as “linear” in the table), which was very close to the performance with a DNN classifier. The omission of the classifier may be interpreted as an additional benefit obtained by the careful design of the prior distribution for the CAAE.

## 4. Discussion

The distribution of the learned latent vectors extracted by a CAAE for the training and test sets with acoustic features are shown in Figure 4a,b, respectively. We can see that the discriminant power of the two-dimensional latent vectors extracted from only the acoustic features may not be strong enough to determine the emotional class of the given utterance. However, in the case with combined acoustic and lexical features, the adoption of the CAAE to extract the latent low-dimensional representation that was relevant to the emotion recognition provided performance improvement unlike in the case of the acoustic features only or lexical features only. The proposed method showed 76.91% in UAR compared to the DNN-based bottleneck multi-modal emotion recognition model [28], which resulted in the UAR of 74.31%, which was higher than the UARs for other conventional methods, as can be seen in Table 3. Figure 4c,d shows the scatter plot of the latent vectors extracted by the CAAE for the training and test datasets of the IEMOCAP database. In contrast to the case with only acoustic features in which even the training data were not clearly separated, utilizing both acoustic and lexical information seemed to provide enough discriminant power that made a clear clustering according to the emotional classes.

The confusion matrices for the proposed method with a DNN classifier and that with a simple classifier checking signs of the components are shown in Table 4 and Table 5, respectively. It was clear that the performance of the speech emotion recognition without any sophisticated classifier was similar in all classes to that with a DNN classifier. In either of the confusion matrices, the misclassification rates between the “neutral” class and other classes were relatively high, which was not surprising in that “neutral” resided in the center of the circumplex model in Figure 1 and represented that the characteristics of any emotion were not strong enough. Table 6 shows the confusion matrix of the CAAE-based method using acoustic features only, which showed the UAR of 66.19% in Table 1. With only acoustic features, the “neutral” class was more confused with other classes as the acoustic characteristics of “neutral” were in the middle of all emotions. The lexical features, however, labeled the sentences without any emotional words as “neutral”, which biased the decision towards the “neutral” class for some cases, compensating the misclassification in the classifier based on acoustic features. Still, the performance for all four classes was rather similar in contrast to some of the reported papers [17,18,27,33], for which the differences between the highest and lowest detection probabilities among four classes range from 27.1% to 64%, resulting in high UARs.

## 5. Conclusions

In this paper, we proposed a multimodal emotion recognition method that captured underlying emotional characteristics from the joint representation of acoustic and lexical features using a CAAE. The acoustic and lexical features were combined and fed into the CAAE to discover the underlying emotion-related low-dimensional representation, which may be interpreted in the VA space in the circumplex model. With a proper design of the prior distribution in the CAAE, the emotional class could be simply decided according to in which quadrant the latent two-dimensional vector fell. Our experiments on the IEMOCAP dataset showed that the proposed method outperformed all other methods that reported the performances for the same dataset in both WAR and UAR.

## Figures and Tables

**Table 1:** Accuracies for different types of acoustic features. WAR, weighted average recall; UAR, unweighted average recall; LLD, low-level descriptor; BN, bottleneck.

Feature Set	WAR	UAR
IS10 [5]	57.2	59.3
IS13 [7]	57.3	58.6
eGeMAPS [8]	54.7	55.3
LLD + MMFCC [21]	59.3	60.2
[BOW${}_{Cep}$] + Cepstrum + GSV [22]	55.4	-
BN [28]	59.7	61.4
CNN-LSTM-DNN [14]	-	60.23
CTC-LSTM [13]	64.0	65.7
LLD + BN	**64.93**	**67.43**
$CAAE_{LLD+BN}$	63.82	66.19

**Table 2:** Accuracies for different types of lexical features.

Feature Set	WAR	UAR
eVector + BOW [22]	58.5	-
mLRF [23]	63.8	64
**v**${}_{BOW}^{utt}$ [21]	63.52	64.55
**v**${}_{w2v}^{utt}$ + **v**${}_{al}^{utt}$ [28]	**64.8**	**65.7**
$v_{w2v}^{utt}+v_{BOW}^{utt}+v_{al}^{utt}$	63.91	64.84
$CAAE_{v_{w2v}^{utt}+v_{BOW}^{utt}+v_{al}^{utt}}$	64.05	64.48

**Table 3:** Accuracies for the multimodal emotion recognition methods.

Feature Set	Classifier	WAR	UAR
$LLD+MMFCC+BOW_{Lex}$ [21]	SVM	69.5	70.1
$LLD+BOW_{Cep}+GSV+eVector+BOW$ [22]	SVM	69.2	-
LLD+mLRF [23]	SVM	67.2	67.3
Hierarchical Attention Fusion [25]	DNN	72.7	72.7
$BN+v_{w2v}^{utt}+v_{al}^{utt}$ [28]	DNN	72.34	74.31
$LLD+BN+v_{w2v}^{utt}+v_{BOW}^{utt}+v_{al}^{utt}$	DNN	72.92	75.44
$CAAE_{LLD+BN+v_{w2v}^{utt}+v_{BOW}^{utt}+v_{al}^{utt}}$ (Proposed)	DNN	**74.37**	**76.91**
$CAAE_{LLD+BN+v_{w2v}^{utt}+v_{BOW}^{utt}+v_{al}^{utt}}$ (Proposed)	linear	**74.08**	**76.72**

**Table 4:** Confusion matrix for the proposed model corresponding to Figure 4 with a DNN classifier (WAR 74.37%, UAR 76.91%).

	**PREDICTION**				
**TRUE**		**ANG**	**HAP**	**NEU**	**SAD**
	**ANG**	85.35	6.82	5.65	2.17
	**HAP**	6.81	79.16	11.53	2.48
	**NEU**	7.22	17.13	63.63	12.02
	**SAD**	2.01	3.07	15.4	79.51

**Table 5:** Confusion matrix for the proposed model corresponding to Figure 4 with a simple classifier checking the signs (WAR 74.08%, UAR 76.72%).

	**PREDICTION**				
**TRUE**		**ANG**	**HAP**	**NEU**	**SAD**
	**ANG**	84.94	6.55	6.08	2.4
	**HAP**	6.86	77.82	12.48	2.85
	**NEU**	8.02	16.22	63.78	11.98
	**SAD**	2.06	4.27	13.39	80.29

**Table 6:** Confusion matrix for ${CAAE}_{LLD+BN}$ in Table 1 only with acoustic features (WAR 63.82%, UAR 66.19%).

	**PREDICTION**				
**TRUE**		**ANG**	**HAP**	**NEU**	**SAD**
	**ANG**	71.84	15.3	10.63	2.22
	**HAP**	10.71	65.93	19.54	3.82
	**NEU**	5.55	24.66	57.58	12.2
	**SAD**	1.88	8.32	20.36	69.45
